# CD8 regulatory T cells are a novel type regulatory T cells in the induction of transplantation immune tolerance

**DOI:** 10.3389/fimmu.2026.1829051

**Published:** 2026-05-05

**Authors:** Jin-can Huang, Yang Zhao, Yu-di Han, Zheng Xia, Guo-sheng Du, Ren Lang, Lin Zhou

**Affiliations:** 1 Devision of Hepatobiliary and Pancreaticosplenic Surgery, Department of General Surgery, Beijing Chao-Yang Hospital, Capital Medical University, Beijing, China; 2 Department of Plastic and Reconstructive Surgery, The First Medical Center, Chinese PLA General Hospital, Beijing, China; 3 Zhantansi Department, Central Medical Branch of PLA General Hospital, Beijing, China; 4 Organ Transplantation Center, General Hospital of Northern Theater Command, Shenyang, China

**Keywords:** dendritic cells, Foxp3, immune tolerance, regulatory T cells, transplantation

## Abstract

**Objective:**

Regulatory T cells (Tregs) play an essential role in transplantation immune tolerance. Although CD4^+^ Tregs have been extensively studied, the contribution of CD8^+^ regulatory T-cell subsets to transplant tolerance remains less well defined. This study aimed to characterize Treg subsets associated with immune tolerance in a rat liver transplantation model and in liver transplant recipients.

**Methods:**

Mesenchymal stem cell-derived tolerogenic dendritic cells (tolDCs) were adoptively infused in a rat liver transplantation model. The frequencies of CD4^+^Foxp3^+^ Tregs and CD8^+^CD45RC^low/-^ Tregs in peripheral blood, spleen, and graft tissue were analyzed by flow cytometry and multiplex immunofluorescence. Peripheral blood samples from liver transplant recipients with long-term stable graft function or acute rejection were also analyzed.

**Results:**

Adoptive infusion of tolDCs significantly prolonged graft survival in the rat liver transplantation model. Increased frequencies of CD4^+^Foxp3^+^ Tregs and CD8^+^CD45RC^low/-^ Tregs were observed in peripheral blood, spleen, and graft tissue in tolDC-induced and spontaneous tolerant animals. MHC-II^+^CD8^+^CD45RC^low/-^ Tregs were enriched within tolerant grafts, suggesting an association with local immune regulation. In liver transplant recipients, the frequency of peripheral CD8^+^CD45RC^low/-^ Tregs was higher in patients with long-term stable graft function than in patients with acute rejection

**Conclusion:**

CD8^+^CD45RC^low/-^ Tregs are associated with transplantation immune tolerance and may cooperate with CD4^+^Foxp3^+^ Tregs in maintaining a tolerant immune microenvironment.

## Introduction

The management of patients with failing organs has been transformed by organ transplantation, which was considered to be the greatest medical advance of this century (1). It has long been acknowledged in the clinic that better treatment outcomes for grafts will result from substituting immunosuppressive medications, either completely or partially, by utilizing tractable physiological processes of self-tolerance (2). How to accomplish this goal is clarified by the more recent finding of a fraction of T-cells that regulate or decrease immunity (3). Adoptive cell therapy in liver transplantation still faces substantial challenges, although some preliminary trials showing that complete drug weaning is possible following adoptive therapy using Treg-enriched cells (4).

More recently, the high expression of Foxp3 is considered essential for the induction and maintenance of immune tolerance with Treg. Further, TGF-β has been demonstrated to significantly influence Foxp3 expression, with studies indicating that TGF-β-dependent pTreg have a role in Treg-dependent experimental allograft tolerance (5, 6). Although CD4^+^Treg cells are the most intensively studied immune regulatory cells in the field of transplantation immune tolerance, CD4^+^Tregs expressing Foxp3 are not a homogeneous component (7). Increasing evidence suggests that CD8^+^ regulatory T-cell subsets may also participate in the induction and maintenance of transplant tolerance. In rat liver transplantation, CD8^+^CD45RC^low/-^ Tregs have been linked to the establishment of a tolerant state. However, the relative distribution and potential contribution of CD4^+^Foxp3^+^ Tregs and CD8^+^CD45RC^low/-^ Tregs across tolerant and rejecting conditions remain insufficiently characterized (8).

However, there are few studies on the role of different components of CD4^+^Treg and CD8^+^Treg in immune tolerance among different classifications of Treg. To find out the relationship between Foxp3 expression, CD8^+^Treg, and CD45RC^low/-^ expression CD4^+^Treg associated with tolerance, and which subgroup is more likely to produce donor antigen-specific Treg, is helpful to clarify the transplantation immune tolerance regulatory network that regulates multiple T cell interactions.

Despite our limited understanding of the antigens that optimally stimulate Treg and their interactions with tissues for suppression, it is noteworthy how extensively efforts have been made to utilize them in therapeutic applications.

## Materials and methods

### Animals

Brown Norway (BN, RT1n) and Lewis (RT11) male rats at the age of 8–10 weeks and a weight of 280-300g were selected (Beijing Viton Lihua Animal Co. LTD, China). Animal experiments received approval from the Animal Experiments and Experimental Animal Welfare Committee of Capital Medical University (No. AEEI-2021-147). Animals were maintained in specified pathogen-free conditions inside a controlled environment featuring a 12-hour light/dark cycle and managed in compliance with the Animal Welfare Act and Institutional Guidelines for the Care and Use of Laboratory Animals.

### Rat model of liver transplantation

Isoflurane (RWD, China) was used as the inhalational anesthetic and administered at 2% in oxygen with a carrier gas flow rate of 0.8–1.0 L/min for anesthesia induction. Pentobarbital sodium (Sigma-Aldrich, USA) was used as the injectable anesthetic and administered intraperitoneally at an initial dose of 40 mg/kg body weight, with supplementary intraperitoneal doses of 10–15 mg/kg given as needed to maintain a surgical plane of anesthesia. Anesthetic depth was monitored throughout the procedure by assessing the pedal withdrawal reflex and respiratory pattern. At the experimental endpoint, animals were euthanized by intravenous injection of 10% potassium chloride at a dose of 0.75–1.0 mL/kg body weight while under deep anesthesia.

An acute rejection (AR) model was established by orthotopic liver transplantation from Lewis donors to BN recipients using a modified double-sleeve technique. A spontaneous tolerance (STOL) model was established by orthotopic liver transplantation from BN donors to Lewis recipients without additional intervention. Rats receiving adoptive infusion of mesenchymal stem cell-derived tolerogenic dendritic cells were assigned to the tolDC group. Animals were therefore divided into three groups: AR, STOL, and tolDC. Peripheral blood and tissue samples were collected on day 7 in the AR group, at the indicated follow-up time points in the tolDC group, and after day 100 in the STOL group.

### HE staining

Transplanted liver tissues were fixed in formalin, embedded in paraffin, sectioned at 5 μm, and stained with hematoxylin and eosin (H&E) for histopathological evaluation. Representative images were acquired by light microscopy.

### Multiparameter flow cytometry

Single-cell suspensions were prepared from peripheral blood, graft tissue, and spleen. Cells were stained with antibodies against CD4, CD8, CD25, Foxp3, and CD45RC and analyzed by multiparameter flow cytometry. Treg subsets were defined as CD4^+^Foxp3^+^CD25^+^ Tregs, CD8^+^Foxp3^+^CD25^+^ Tregs, CD4^+^CD45RC^low/-^ Tregs, and CD8^+^CD45RC^low/-^ Tregs.

### Multiplex immunofluorescence assay

CD4^+^ and CD8^+^Tregs in the liver graft of multiplex immunofluorescent staining were stained with the Opal 7 color Manual IHC kit (PerkinElmer, Hopkinton, MA, USA). The steps before incubation with the first antibodies were consistent with those of the aforementioned IHC staining. Tissues were incubated with antibodies of CD4, Foxp3, CD8, CD45RC, and MHC-II serially for 1 hour and further labeled with the corresponding Opal Fluorophore for a 10-minutes to visualize the combined antibodies. Then the tissue sections were subsequently tagged with anti-rabbit/mouse Polymeric Horseradish Peroxidase (PerkinElmer, Hopkinton, MA, USA) for a duration of 10 minutes. Finally, the sections were subsequently counterstained with spectral DAPI for 10 minutes, mounted using an anti-fade medium (P36965, Life Technologies), and preserved at 4°C prior to comprehensive scanning with the Vectra Polaris multispectral imaging platform (Akoya Biosciences), during which 5–7 images of notable regions were captured for further analysis. Image analysis was conducted utilizing the In Form 2.4.8 Image Analysis Software (Akoya Biosciences).

### Clinical cohort

Peripheral blood samples were collected from liver transplant recipients treated at Beijing Chao-Yang Hospital between January 2018 and December 2022. Patients were classified into the long-term stable graft function group or the acute rejection group according to predefined clinical and pathological criteria, as summarized in **Supplementary Table 1**. Flow cytometric analysis was performed to determine the frequency of peripheral CD8^+^CD45RC^low/-^ Tregs.

### Statistical analysis

Statistical analyzes were performed using GraphPad Prism 10.1.2. Data are presented as mean ± SD unless otherwise specified. Comparisons among multiple groups were performed using one-way ANOVA, and comparisons between two groups were performed using the unpaired Student’s t-test. Categorical variables were compared using the χ² test. Survival curves were generated using the Kaplan-Meier method and compared using the log-rank test. A two-sided P value < 0.05 was considered statistically significant.

## Results

### Spontaneous tolerance and tolDC-induced tolerance models of liver transplantation were successfully established in rats

**Figure 1:**
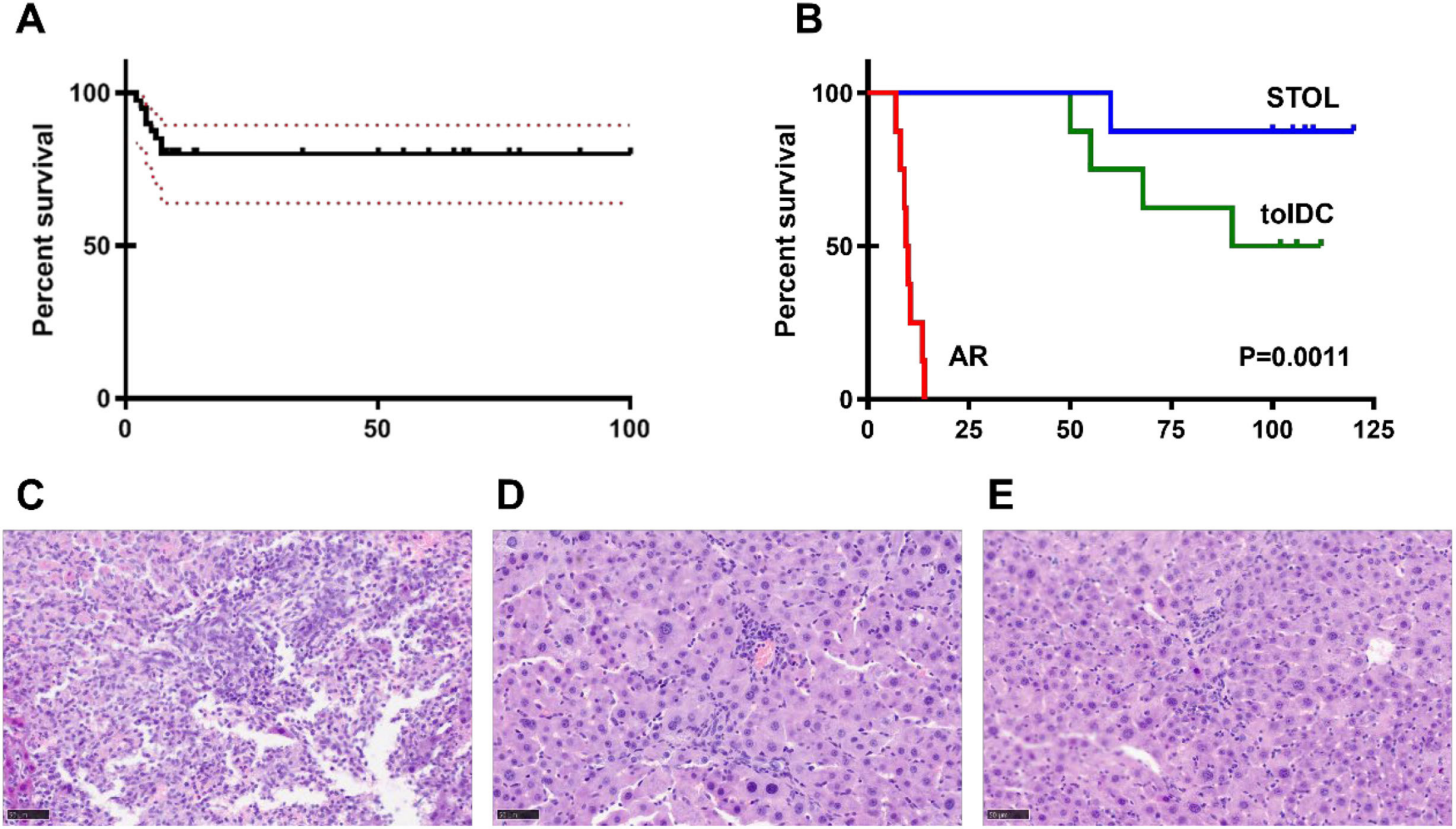
Establishment of different types of rat liver transplantation models, n=6. The success rate of model establishment using the modified double-sleeve technique exceeded 95% **(A)**. Survival was significantly prolonged in tolDC rats compared with untreated AR rats **(B)**. Representative H&E staining of graft tissues from the different groups is shown in **(C–E)**.

An acute rejection model was established by liver transplantation from Lewis donors to BN recipients, whereas a spontaneous tolerance model was established by transplantation from BN donors to Lewis recipients. Six rats were included in each experimental group. The overall success rate of the model was greater than 95% (**Figure 1A**). Compared with untreated AR rats, AR rats receiving tolDC infusion showed significantly prolonged survival (**Figure 1B**). The median survival time was 10 days in the AR group, whereas it was not reached during the observation period in the tolDC group and the STOL group. All models were confirmed by pathological findings (**Figures 1C–E**).

### Increased frequencies of CD8^+^CD45RC^low/-^ Tregs were associated with transplantation immune tolerance

**Figure 2:**
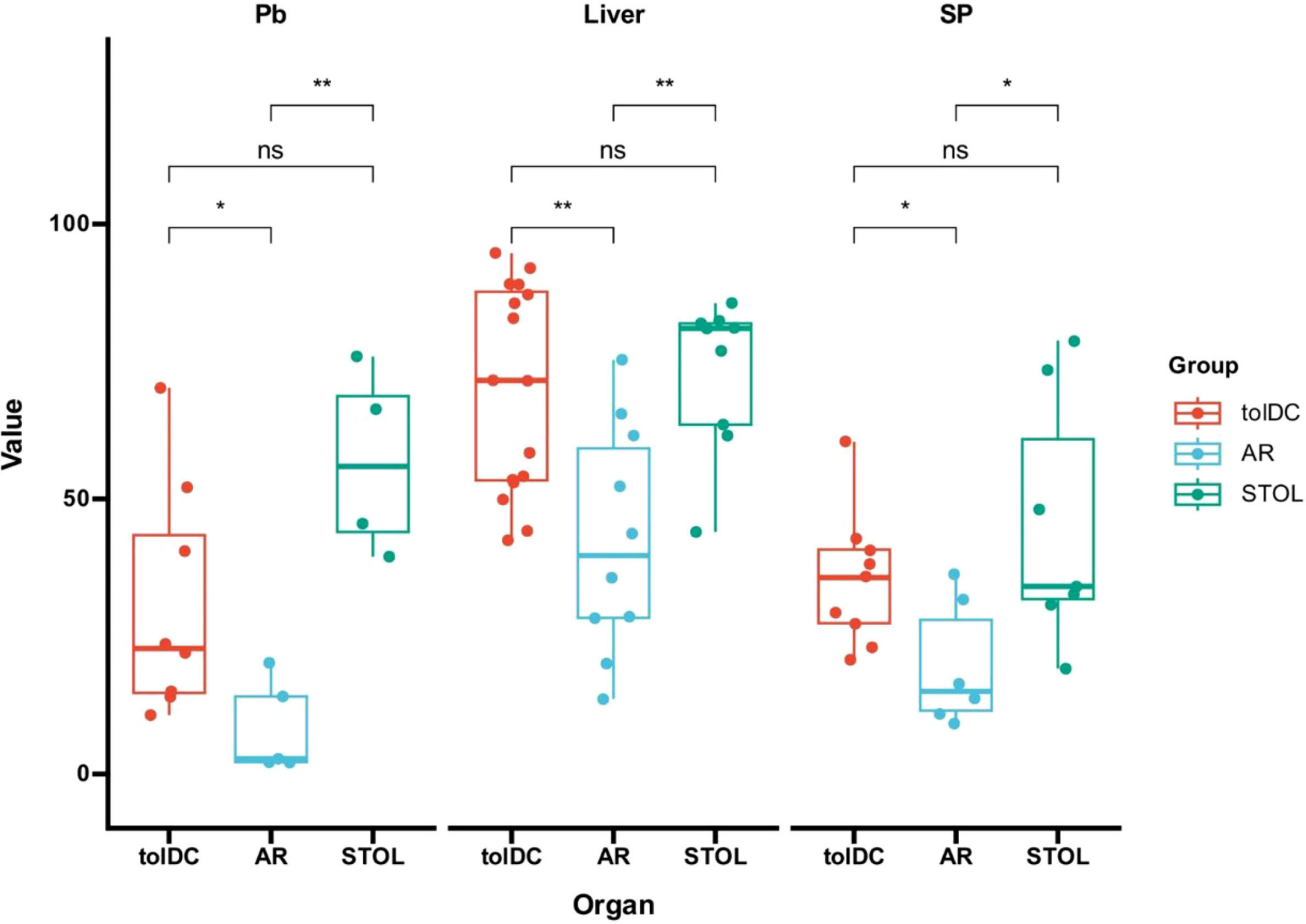
Increased frequencies of CD8^+^CD45RC^low/-^ Tregs were associated with transplantation immune tolerance (n ≥ 3). Compared with AR rats, tolDC and STOL rats showed increased proportions of CD8^+^CD45RC^low/-^ Tregs in peripheral blood, liver grafts, and spleens. Further comparison between the tolDC and STOL groups showed no significant difference in liver grafts or spleens, with only a marginal difference in peripheral blood. AR, acute rejection; STOL, spontaneous tolerance; Pb, peripheral blood; SP, spleen. *P < 0.05, **P < 0.01; ns, not significant.

Long-term graft survival in rats was associated with increased frequencies of CD8^+^CD45RC^low/-^ Tregs in graft tissue. Compared with AR rats, tolDC and STOL rats showed significantly higher proportions of CD8^+^CD45RC^low/-^ Tregs in peripheral blood, liver grafts, and spleens (**Figures 2A–C**). Further comparison between the tolDC and STOL groups showed no significant difference in liver grafts or spleens, with only a marginal difference in peripheral blood.

### The high expression of Foxp3^+^Treg contributes to the establishment of immune tolerance

**Figure 3:**
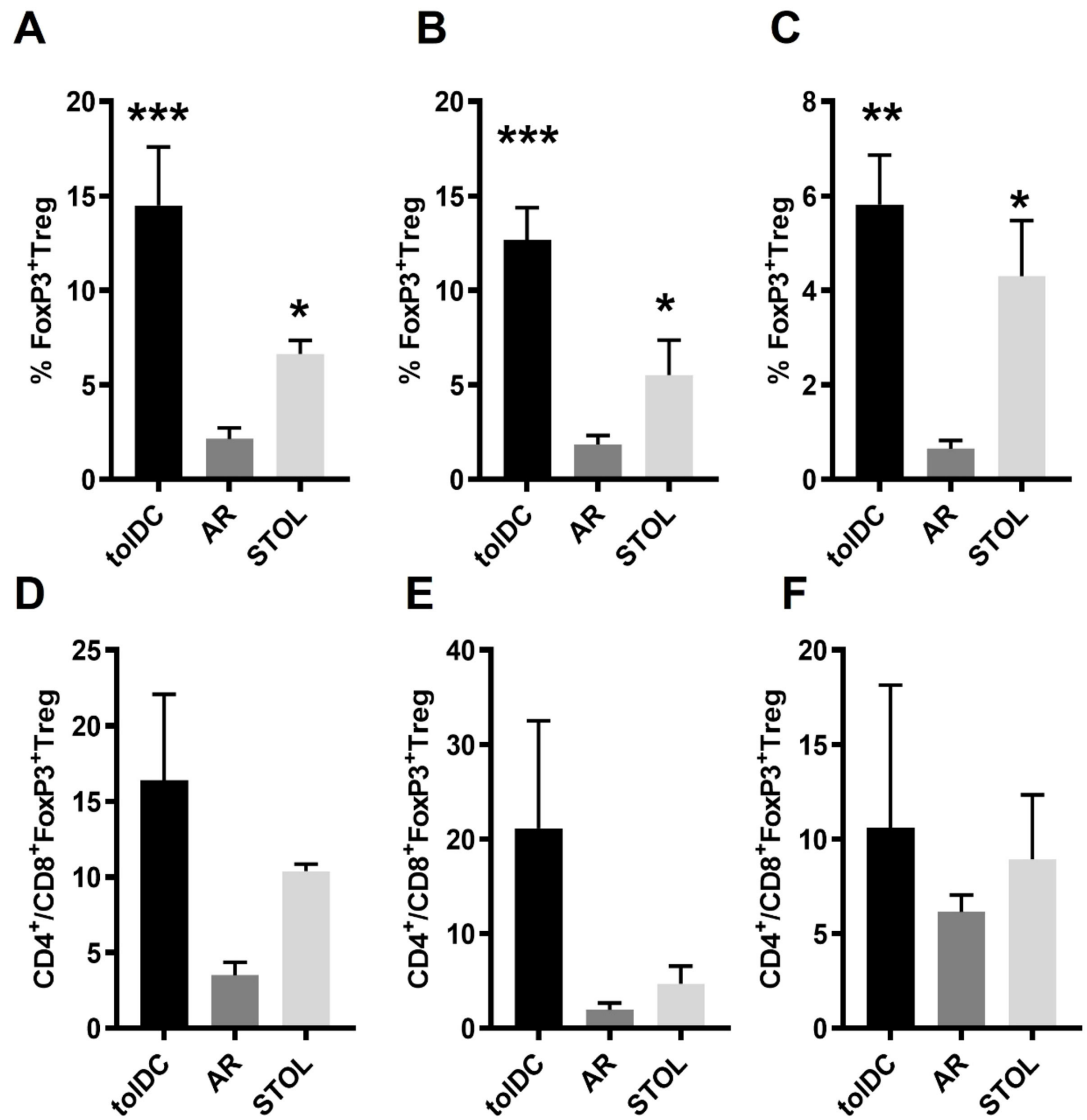
Increased Foxp3^+^ Tregs were associated with transplantation immune tolerance (n ≥ 3). Compared with AR rats, tolDC and STOL rats showed increased proportions of Foxp3^+^ Tregs in peripheral blood **(A)**, liver grafts **(B)**, and spleens **(C)**. Further analysis of CD4^+^Foxp3^+^ and CD8^+^Foxp3^+^ subsets showed that the CD4^+^ subset predominated over the CD8^+^ subset **(D–F)**, with the highest ratio observed in liver grafts from tolDC rats **(E)**. AR, acute rejection; STOL, spontaneous tolerance. *P < 0.05, **P < 0.01, ***P < 0.001.

Foxp3^+^CD25^+^Treg expression in peripheral blood, transplanted liver and spleen of different rats indicated significant differences among groups (P < 0.01). Compared with the AR group, Foxp3^+^CD25^+^Treg was highly expressed in peripheral blood, transplanted liver, and spleen of tolDC infusion induced and spontaneous tolerance rats (**Figures 3A–D**). Further analysis of CD4 and CD8 subsets in Foxp3^+^CD25^+^Treg showed that the expression of CD4^+^Foxp3^+^Treg was the main proportion in both spontaneous tolerance rats, adoptive tolDC rats, and AR rats. These changes in peripheral blood, transplanted liver, and spleen were consistent.

**Figure 4:**
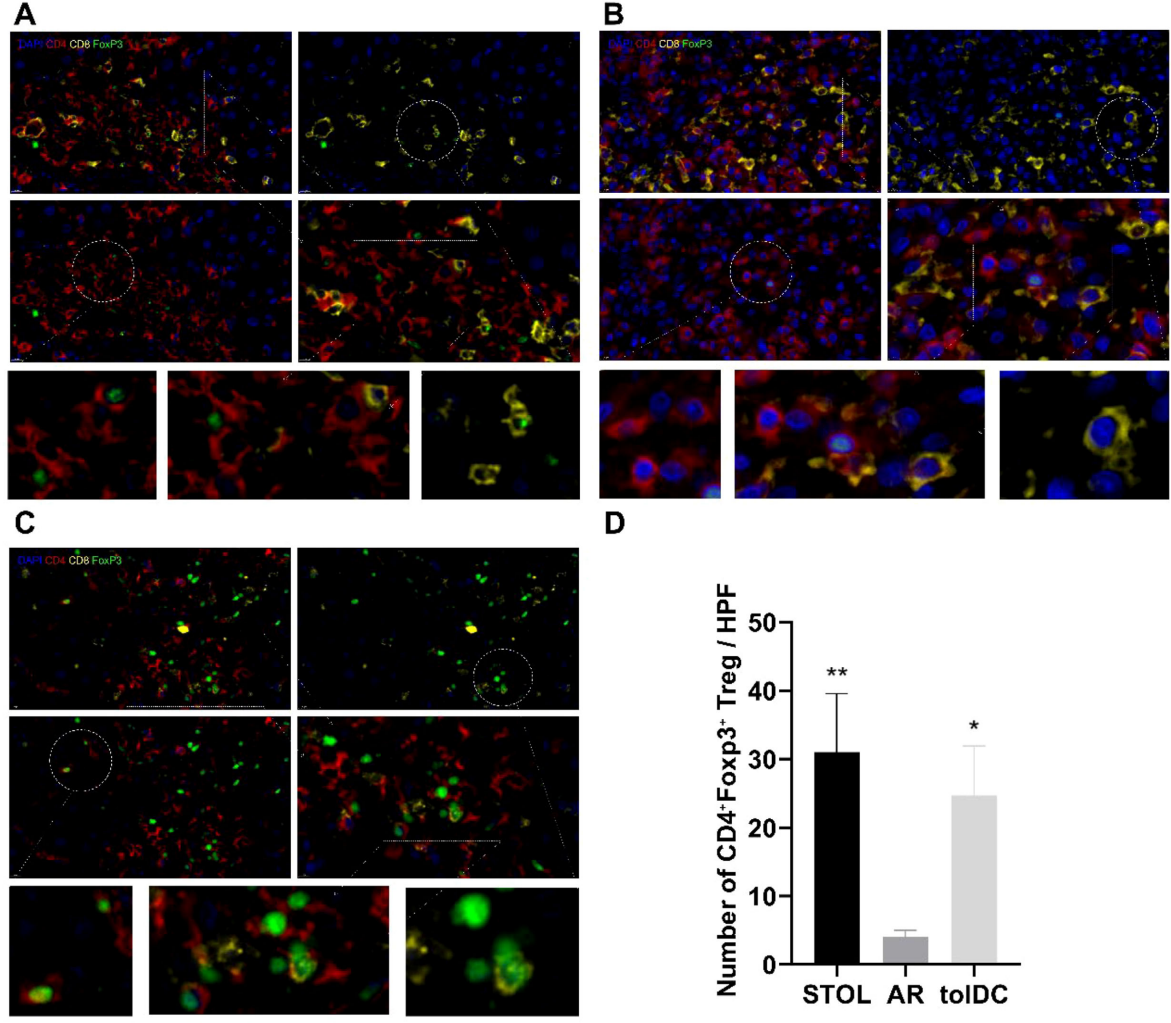
Expression of CD4^+^Foxp3^+^ Treg in different rat liver transplantation models. Multiplex immunofluorescence staining shows the expression of CD4 (red), CD8 (yellow), and Foxp3 (green), with DAPI used for nuclear staining (blue). Compared with AR rats **(B)**, CD4^+^Foxp3^+^ Tregs were more abundant in graft tissues from STOL rats **(A)** and tolDC rats **(C)**, as indicated by the dashed white circles and enlarged insets. Quantitative analysis confirmed that the number of CD4^+^Foxp3^+^ Treg cells was significantly higher in STOL and tolDC rats than in AR rats **(D)**. *P < 0.05, **P < 0.01.

The level of CD4^+^Foxp3^+^Treg was up to 20 times higher than that of CD8^+^Foxp3^+^Treg, which appeared in liver grafts of adoptive tolDC rats (**Figures 3E, F**). Univariate ANOVA showed no difference in the proportion of different transplanted rats and different tissue sites, and no significant difference within the group. The expression of CD4^+^Foxp3^+^Treg in transplanted liver was also confirmed by multi-color immunofluorescence staining (**Figure 4**).

### Enrichment of MHC-II^+^ CD8^+^CD45RC^low/-^ Tregs was observed in tolerant liver grafts

**Figure 5:**
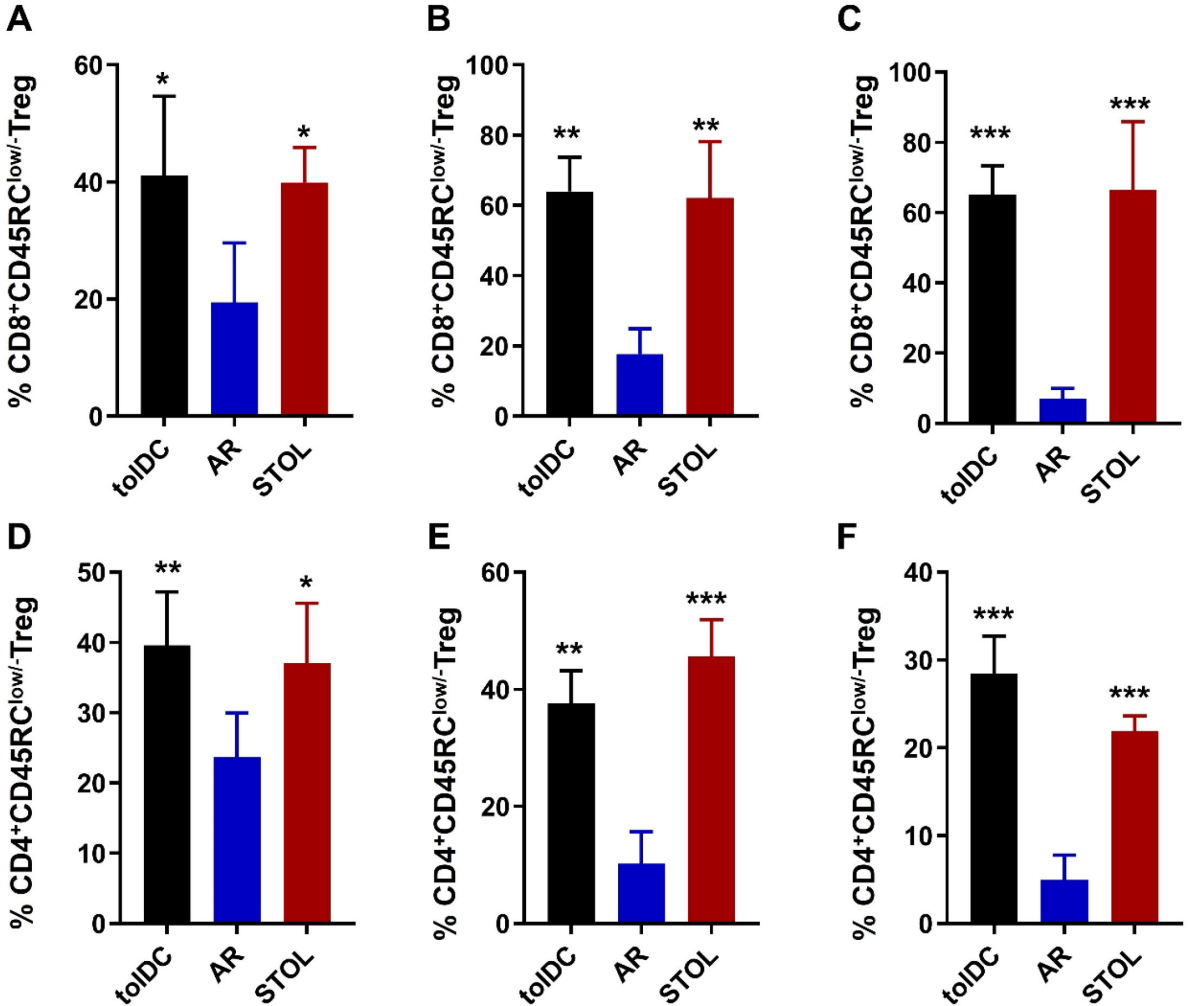
Increased proportions of CD45RC-defined regulatory T-cell subsets were associated with immune tolerance induction. Compared with AR rats, tolDC and STOL rats showed increased proportions of CD8^+^CD45RC^low/-^ Tregs and CD4^+^CD45RC^low/-^ Tregs in peripheral blood **(A, D)**, liver grafts **(B, E)**, and spleens **(C, F)**. AR, acute rejection; STOL, spontaneous tolerance. *P < 0.05, **P < 0.01, ***P < 0.001.

**Figure 6:**
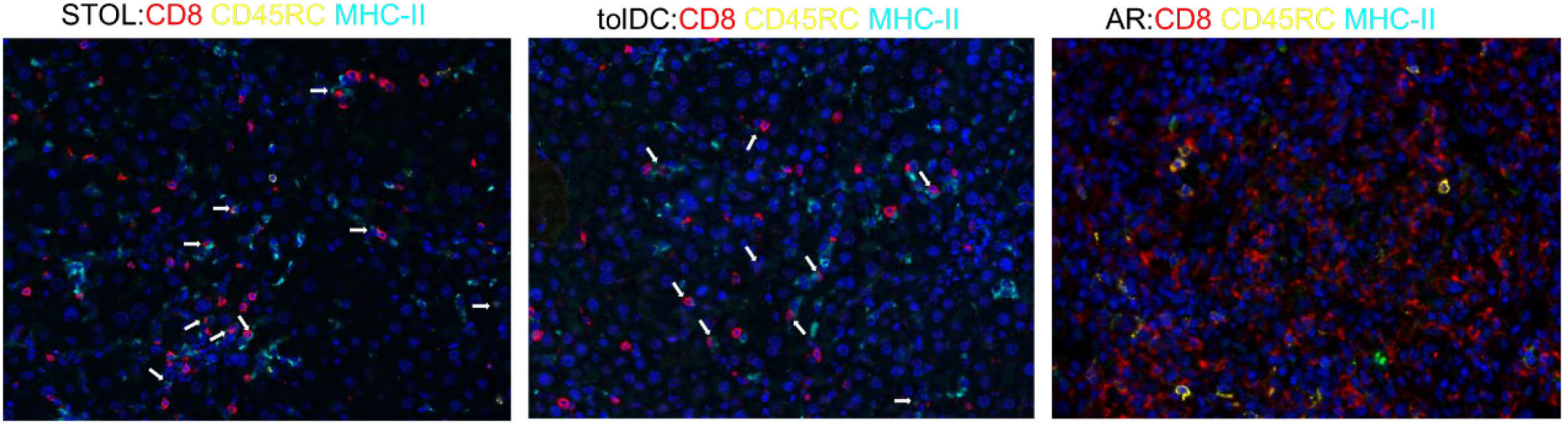
Enrichment of MHC-II^+^ CD8^+^CD45RC^low/-^ Tregs in tolerant liver grafts. Multiplex immunofluorescence staining shows the expression of CD8 (red), CD45RC (yellow), and MHC-II (cyan). Compared with AR rats, tolDC and STOL rats showed increased numbers of MHC-II^+^ CD8^+^CD45RC^low/-^ Tregs in graft tissue, as indicated by the white arrows. AR, acute rejection; STOL, spontaneous tolerance.

The frequency of CD8^+^CD45RC^low/-^ Tregs in peripheral blood, transplanted liver tissue, and spleen was markedly elevated in tolDC-induced and spontaneous tolerant rats compared with AR rats (**Figures 5A–C**). Multiplex immunofluorescence further demonstrated enrichment of MHC-II^+^ CD8^+^CD45RC^low/-^ Tregs within tolerant liver grafts (**Figure 6**).

### CD4^+^CD45RC^low/-^ Tregs were also increased in tolerant animals

CD4^+^CD45RC^low/-^ Tregs were increased in peripheral blood, transplanted liver tissue, and spleen in tolDC-induced and spontaneous tolerant rats compared with AR rats (**Figures 5D–F**). Although one-way ANOVA indicated significant differences among groups, no significant difference was observed between the tolDC-induced and spontaneous tolerance groups. The ratio of CD8^+^CD45RC^low/-^ Tregs to CD4^+^CD45RC^low/-^ Tregs ranged from approximately 2-fold to 6-fold and was highest in transplanted liver tissue, suggesting that CD8^+^CD45RC^low/-^ Tregs may represent a prominent CD45RC-defined regulatory subset in the tolerant setting.

### Higher frequencies of peripheral CD8^+^CD45RC^low/-^ Tregs were detected in recipients with long-term stable graft function

**Figure 7:**
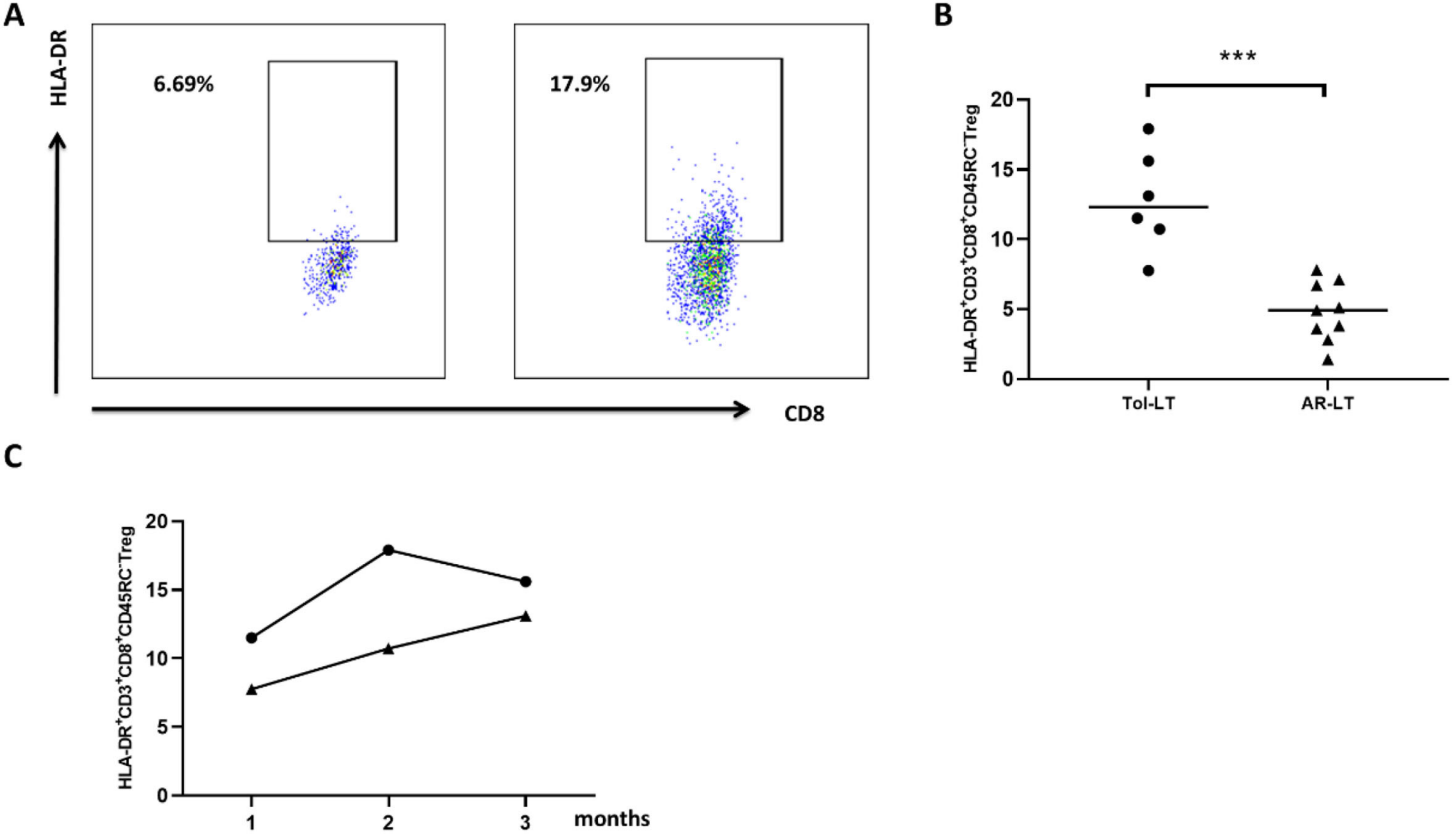
Increased peripheral CD8^+^CD45RC^low/-^ Treg levels were associated with long-term stable graft function after liver transplantation. Patients with long-term stable graft function showed significantly higher levels of peripheral CD8^+^CD45RC^low/-^ Tregs than patients with acute rejection **(A, B)**; long-term stable group, n = 6; acute rejection group, n = 9). In addition, early postoperative monitoring showed relatively low levels of CD8^+^CD45RC^low/-^ Tregs in recipients who developed acute rejection **(C)** n = 3). ***P < 0.001.

Peripheral blood analysis of liver transplant recipients showed that patients with long-term stable graft function had a significantly higher frequency of CD8^+^CD45RC^low/-^ Tregs than patients with acute rejection. The analyzes shown in **Figures 7A, B** included six patients in the long-term stable graft function group and nine patients in the acute rejection group. In addition, early postoperative monitoring in three recipients suggested that patients who developed acute rejection exhibited relatively low levels of CD8^+^CD45RC^low/-^ Tregs (**Figure 7C**).

## Discussion

The present study demonstrates that mesenchymal stem cell-derived tolerogenic dendritic cells are associated with the induction of transplantation immune tolerance in a rat liver transplantation model. In tolerant animals, both CD4^+^Foxp3^+^ Tregs and CD8^+^CD45RC^low/-^ Tregs were enriched in peripheral blood, lymphoid tissues, and grafts. These findings support the concept that transplantation tolerance is maintained by coordinated regulation among multiple Treg subsets rather than by a single regulatory population (9, 10).

With tolDC treatment, we observed increased frequencies of CD8^+^CD45RC^low/-^ Tregs and CD4^+^Foxp3^+^ Tregs in peripheral blood, grafts and spleen. These findings are consistent with the possibility that tolDC treatment promotes a regulatory immune milieu and supports tolerance-associated Treg expansion. However, because direct *in vitro* suppression assays were not presented in the current study, the functional consequences of these phenotypic changes should be interpreted with caution.

Our data indicate that CD8^+^CD45RC^low/-^ Tregs are enriched in tolerant grafts and may contribute to the establishment of a tolerant immune microenvironment. These findings support prior reports that CD8^+^ Tregs can exert immunoregulatory functions independently of Foxp3 expression (11–13). Zhou et al. also demonstrated that CD8^+^CD45RC^low/–^ Tregs foster tolerance in rat liver transplantation through Foxp3−independent mechanisms (14). In the present study, however, no direct head-to-head functional comparison between CD8^+^CD45RC^low/-^ Tregs and CD4^+^Foxp3^+^ Tregs was performed. Therefore, our results support an association with tolerance rather than a definitive conclusion regarding functional superiority.

In our models, CD8^+^CD45RC^low/–^ Tregs displayed elevated MHC-II expression within tolerant grafts. Tolerogenic dendritic cells are increasingly viewed as important contributors to transplantation tolerance, partly through reduced co-stimulatory signaling, altered antigen presentation, and support of regulatory T-cell responses (15). In this setting, the enrichment of MHC-II^+^ CD8^+^CD45RC^low/–^ Tregs may be relevant. One possible explanation is trogocytosis, a process by which immune cells acquire membrane-associated molecules from interacting cells. Previous studies suggest that dendritic cells can transfer MHC-II molecules to CD8^+^ regulatory T cells through this mechanism (16–19). Although trogocytosis was not directly tested in the present study, the increased MHC-II expression in graft-infiltrating CD8^+^CD45RC^low/–^ Tregs is consistent with, but does not prove, this possibility.

CD4^+^Foxp3^+^ Tregs also appeared to play important roles in the tolerant state. They constituted the major Foxp3^+^ subset and were consistently increased in tolerant animals. The coexistence of increased CD4^+^Foxp3^+^ Tregs and CD8^+^CD45RC^low/-^ Tregs highlights the complexity of immune regulation in transplantation, in which multiple regulatory subsets may act in parallel or cooperatively to maintain tolerance (20). Previous studies suggest that CD4^+^Foxp3^+^ Tregs participate in tolerance induction through TGF-β-related pathways, whereas CD8^+^ regulatory T-cell subsets may contribute to sustained regulation (21, 22). In our study, tolDC infusion was associated with increases in both subsets, supporting the possibility that tolDCs help shape a tolerance-favoring immune environment.

Clinically, these findings are promising. We detected higher frequencies of CD8^+^CD45RC^low/–^ Tregs in long−term liver transplant survivors than in patients with acute rejection. This supports the clinical relevance of Treg−based therapies (23, 24). These results align with recent trials showing that Treg adoptive transfer can reduce rejection and enable immunosuppressive drug withdrawal (25, 26). Although the clinical cohort was limited in size, this finding supports the translational relevance of tolerance-associated regulatory T-cell subsets in liver transplantation. Together with previous studies on Treg-based cellular therapy and regulatory dendritic cell therapy, our results suggest that tolDC-based approaches may provide a platform for promoting donor-specific immune regulation (27–29).

Nevertheless, our study has limitations. First, the current manuscript primarily provides phenotypic and distributional evidence and does not include direct functional suppression assays comparing CD8^+^CD45RC^low/-^ Tregs and CD4^+^Foxp3^+^ Tregs. Second, the molecular basis of MHC-II acquisition by CD8^+^CD45RC^low/-^ Tregs was not directly examined in this study. Third, the clinical cohort size was limited. Future studies should include dedicated suppression assays, cytokine and checkpoint profiling, mechanistic validation of trogocytosis-related pathways, and larger clinical cohorts.

In summary, our findings suggest that CD8^+^CD45RC^low/-^ Tregs are associated with transplantation immune tolerance, particularly within graft tissues, and may cooperate with CD4^+^Foxp3^+^ Tregs in maintaining a tolerant immune environment.

## Data Availability

The raw data supporting the conclusions of this article will be made available by the authors, without undue reservation.
