# Supplementary material for: CD8 regulatory T cells are a novel type regulatory T cells in the induction of transplantation immune tolerance
Source: Front Immunol. 2026 May 5;17:1829051. doi: 10.3389/fimmu.2026.1829051 (PMC13183572; doi:10.3389/fimmu.2026.1829051)
Supplement: Supplementary file 1 [file Table1.docx]

**Supplementary Table 1. Clinical characteristics and group definitions of liver transplant**

| **Group** | **n** | **Age (years)** | **Sex (M/F)** | **Time after transplantation** | **Immunosuppressive regimen** | **Criteria** |
| --- | --- | --- | --- | --- | --- | --- |
| long-term stable graft function | 6 | 48.7 ± 9.6 | 4/2 | 38.5 ± 11.2 months | Tac-based maintenance therapy with or without MMF/Pred | Stable liver function, no clinical evidence of rejection during follow-up |
| acute rejection | 9 | 46.9 ± 10.4 | 6/3 | 2.9 ± 1.5 months | Tac-based maintenance therapy with or without MMF/Pred | Acute rejection diagnosed by clinical, biochemical, and/or histopathological criteria |
